# Mental calculation increases physiological postural tremor, but does not influence physiological goal-directed kinetic tremor

**DOI:** 10.1007/s00421-022-05039-6

**Published:** 2022-09-19

**Authors:** Francesco Budini, Richard Mocnik, Markus Tilp, Domenico Crognale

**Affiliations:** 1 Institute of Human Movement Science, University of Graz, Sport and Health, Karl-Franzens-Universität Graz, Mozartgasse 14/I (Sportwissenschaft), 8010 Graz, Austria; 2 Institute for Sport and Health, University College of Dublin, Dublin, Ireland

**Keywords:** Physiological tremor, Hand postural tremor, Goal-directed tremor, Mental calculation, Cognitive effort, Hand dexterity

## Abstract

**Purpose:**

During a cognitive effort, an increase in cortical electrical activity, functional alterations in the anterior cingulate cortex, and modifications in cortical inputs to the active motor units have been reported. In light of this, an increase in tremor could be anticipated as result of a mental task. In the present work, we tested this hypothesis.

**Methods:**

In 25 individuals, tremor was measured with a three-axial accelerometer during 300 s of postural and goal-directed tasks performed simultaneously to mental calculation, or during control (same tasks without mental calculation). Hand and finger dexterity were also evaluated. Electromyographic (EMG) recordings from the extensor digitorum communis were collected during the postural task.

**Results:**

Hand and finger dexterity was negatively affected by the mental task (*p* = .003 and *p* = .00005 respectively). During mental calculation, muscle tremor increased in the hand postural (+ 29%, *p* = .00005) but not in the goal-directed task (− 1.5%, *p* > .05). The amplitude of the main frequency peak also increased exclusively in the hand postural task (*p* = .028), whilst no shift in the position of the main frequency peak was observed. EMG was not affected.

**Conclusion:**

These results support the position of the contribution of a central component in the origin of physiological hand postural tremor. It is suggested that the different effect of mental calculation on hand postural and goal-directed tasks can be attributed to the different origins and characteristics of hand postural and goal-directed physiological tremor.

## Introduction

In clinical settings, fatigue is best defined as difficulty in initiation of or sustaining voluntary activities (Chaudhuri and Behan 2004). This effect can be caused by peripheral and central mechanisms within the nervous system (Gandevia 2001; Taylor and Gandevia 2008) as well as cellular mechanisms within the muscles (Fitts 1994). However, it has for long been suggested that one additional component of fatigue is of cognitive nature and it is referred to as mental fatigue (Franz 1897).

Although the concept of mental fatigue as a result of prolonged cognitive activities is uncomplicated and intuitive to understand, its effects on the human body can be challenging to describe in physiological terms. In fact, while the effects of mental fatigue on both cognitive (Lorist et al. 2000; Boksem et al. 2005; Lim and Dinges 2008; Langner et al. 2010; Budini et al. 2014a; Sasahara et al. 2015; Hopstaken et al. 2016) and physical performance (for reviews (Van Cutsem et al. 2017; Pageaux and Lepers 2018; Brown et al. 2020)) have been extensively investigated, the underpinning physiological mechanisms of these effects remain unclear.

Cognitive efforts increase electroencephalographic activity (Lal and Craig 2002; Boksem et al. 2005; Tartaglia et al. 2008; Zhao et al. 2012) and functional alterations have been reported in anterior cingulate cortex in individuals assessed during cognitive tasks (Lorist et al. 2005; Lim et al. 2010). Since the anterior cingulate cortex region of the brain is concerned with motor learning and control, a deterioration in fine movements, dexterity, and force steadiness can be expected. Moreover, an altered glucose metabolism in this region has been observed in tremor related diseases (Ivanov et al. 2015; Schöberl et al. 2017). Accordingly, an increase in force fluctuations was observed in isometric tasks when a cognitive effort was added to the exercise (Lorist et al. 2002; Vanden Noven et al. 2014) and, similarly, cognitive tasks are executed more slowly and less accurately during simultaneous isometric contractions (Zijdewind et al. 2006). Speed-accuracy of goal-directed arm movements (Rozand et al. 2015) and hand dexterity (Duncan et al. 2015; Valenza et al. 2020) also decrease during cognitive effort.

All these results, however, could be attributed to a dual-task (cognitive–motor) interference, since the subjects could volitionally control the physical task they were performing, so they were essentially executing two different tasks simultaneously. On the contrary, muscle tremor, being defined as involuntary rhythmic oscillations during postural or dynamic muscle contractions that is datable in every person (postural and kinetic physiological tremor respectively) (Marshall and Walsh 1956; Deuschl et al. 1998), cannot be cognitively regulated. Nevertheless, being, at least partially, produced by a central component (Vallbo and Wessberg 1993; Bye and Neilson 2010), it could also be affected by a mental effort (Lorist et al. 2005; Lim et al. 2010). Pereira and colleagues (Pereira et al. 2019) attributed the observed decline in force steadiness during the cognitive effort to modifications in cortical inputs to the active motor units performing the task/exercise. In light of this, an increase in tremor could be anticipated as result of mental fatigue. However, recently we could not observe an increase in physiological hand postural or kinetic tremor following the termination of 100 min of continuative cognitive task (Budini et al. 2022). We hypothesised that those neurophysiological alterations commonly observed during mental fatigue (Lal and Craig 2002; Boksem et al. 2005; Tartaglia et al. 2008; Lim et al. 2010; Zhao et al. 2012) that could influence tremor (as alteration in the activity of the anterior cingulate cortex), only persist for the duration of the cognitive task and not inducing therefore any detectable effect after the mental task is terminated.

The aim of the present study is to test whether physiological hand postural and goal-directed kinetic tremor is affected during a cognitive effort.

## Methods

### Participants, study design and procedures

Twenty-five recreationally active individuals (age range 25–50 years): 17 males (31.8 ± 8 years, 76.5 ± 7.1 kg, 182 ± 6 cm) and 8 females (29.8 ± 3 years, 63.3 ± 14 kg, 166 ± 6 cm), with no history of neurological disorders and free from any medication, volunteered for the experiment. Volunteers were required to abstain from any strenuous physical activity on the test day, as well as refraining from taking caffeine-containing substances or smoking in the 2 h period before the test session. The study was approved by the Review Board of the University of Graz (GZ. 39/128/63 ex 2020/21) and written informed consent was obtained from all volunteers before the onset of the experimental procedures.

The participants were requested to attend the laboratory for one single experimental session, lasting about 75 min. Before starting data collection, the volunteers were prepared for surface electromyography (EMG) recording and completed five familiarisation trials for the goal-directed kinetic tremor task (details in the following sections). The experiment consisted in the measurement of hand postural and goal-directed tremor during a cognitive effort (intervention, details in the following sections) or not (control). For each subject, each measurement was therefore performed twice: in the control and the intervention conditions. Tremor was recorded continuously for 300 s using a three-axis accelerometer (MPU–6050, SparkFun Electronics^®^) secured to the dorsal aspect of the hand with the y axis aligned with the third metacarpal bone. All measurements were conducted one after the other in random order. On a subsample of six participants (32.7 ± 8 years, 68.3 ± 14.9 kg, 176 ± 13 cm, 3 males), we additionally tested whether the cognitive effort had a lasting effect. The measurements in this case were performed in a not-randomised order with baseline recordings (control), followed by the recordings during the intervention and recordings immediately after the intervention. On 13 participants, we additionally tested finger dexterity through a Purdue pegboard test.

### Hand postural tremor assessment

**Fig. 1:**
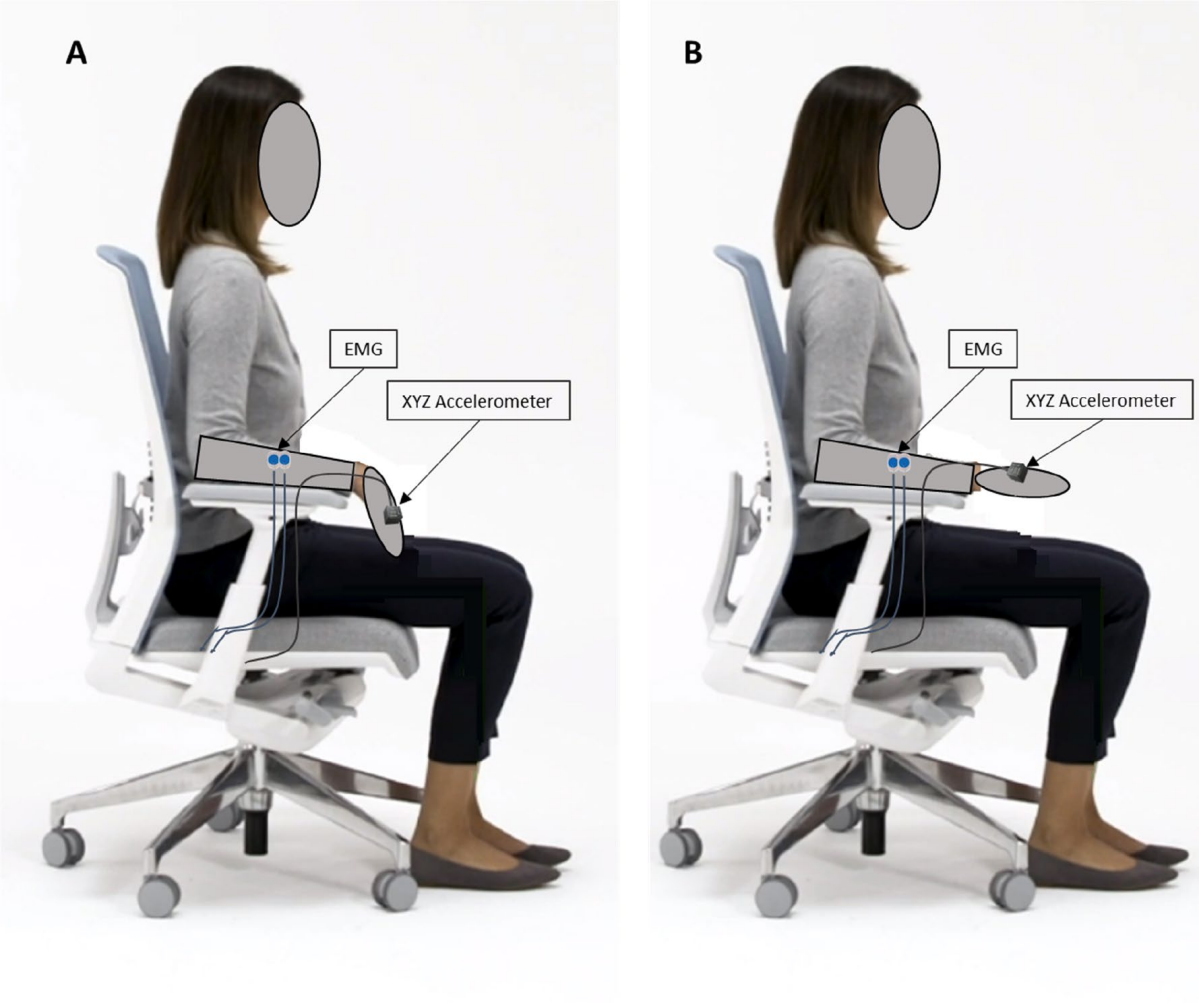
Set and position for the hand postural tremor task. **A**: relaxed position. **B**: hand in line with the elbow and fingers loosely extended

The volunteers seated on a chair with their forearm supported on the armrest and with the wrist joint aligned to its edge, so that the hand was not supported by the armrest; the instruction was to maintain the hand horizontally in prone position and in line with the supported forearm, the fingers loosely extended and to gaze upon a fixed point at 1.5 m distance (Elble 2003) (Fig. 1).

### Goal-directed kinetic tremor and hand dexterity assessment

**Fig. 2:**
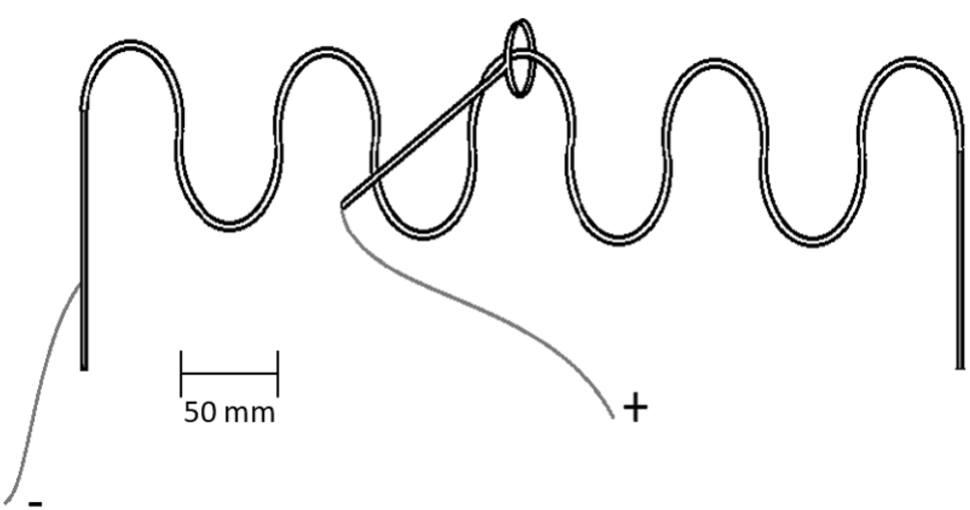
The buzz wire circuit for the goal-directed kinetic tremor assessment

Goal-directed tremor was recorded during 300 s continued performance of a buzz wire circuit: the participant was sitting in front of a 0.5 m long wire comprising five bends of the same size and shape (half-circle ~ 5 cm diameter) while holding a wand (20 g of mass) with a 4 cm diameter metal loop at its top (Fig. 2). The volunteers were required to follow the wire shape with the wand loop engaged in the circuit and complete the circuit from left to right and back, trying not to touch the wire loop with the wand while performing prono-supination movements only (subjects were instructed not to rotate the wand between the fingers). For this task, the volunteers had five familiarisation trials before the beginning of the test session. During the familiarisation trials, the volunteers were invited to find a comfortable posture and a suitable distance from the circuit that allowed the performance of the task without moving on the chair. The volunteers were asked to try to maintain approximately the same pace for both the five familiarisation rounds and the subsequent 300 s continuous test. However, no directions about the execution speed were given and the task was always self-paced. We opted for this test because assessing kinetic tremor during a goal-directed task proved to be effective in highlighting the characteristic tremor frequency components in both pathological and healthy individuals (Budini et al. 2014a, 2017). Acceleration signals were A/D converted, using the Cambridge Electronic Design (CED) Power1401 system, and captured at a sampling rate of 1000 Hz using the CED Spike2 V10 package.

### Purdue pegboard test (finger dexterity)

Thirteen participants (31.8 ± 7 years, 71.8 ± 11.8 kg, 176 ± 10 cm, 9 males) completed a Purdue Pegboard Assembly test to assess finger dexterity (Fleishman and Ellison 1962). The board has four built-in compartments on the top part (two of which we filled with pins, one with washers and one with collars) and two parallel rows of 25 holes. For the finger dexterity task, the volunteers were instructed to take one pin at a time from the built-in compartment of the board, place it into the first hole and proceed sequentially from up to down. Once completed the first row, the task continued by inserting washers in the pins, then by inserting pins also in the second row of holes and placing the collars in the pins. Finally, the volunteers had to remove all the collars and replace them in the built-in compartment. It was required to complete the task as quickly as possible and the total time was recorded.

### Cognitive protocol

For the cognitive exercise, the volunteer was asked to continuously subtract 13 from a three-digit number and verbally report the result of each subtraction within 4 s. In case of a mistake in the calculation or time exceedance, the volunteer had to start again from the beginning. The cognitive tasks were continued for the entire duration of the kinetic and hand postural task, as well as throughout the finger dexterity test.

### Electromyography

After appropriate skin preparation, surface EMG was recorded from the extensor digitorum communis of the dominant arm by adhesive electrodes (size: 44.2 × 22 mm, model: Blue Sensor N, Ambu A/S, Ballerup, Denmark) placed in standard bipolar configuration with 20 mm interelectrode distance and the ground electrode placed over the olecranon process of ulna. EMG signals were A/D converted, using the Cambridge Electronic Design (CED) Power1401 system, and captured at a sampling rate of 2000 Hz using the CED Spike2 V10 package. In order to avoid phase shift, no low pass filter was applied. Limitation of the bandwidth with 60 kHz was determined by the isolation amplifier.

### Data analysis

Data files were stored on a PC and analysed using custom algorithms developed in MATLAB (9.6.0.1072779 R2019a).

The acceleration signal collected during both hand postural and goal-directed kinetic (buzz wire) tremor task was band-pass (2–30 Hz) filtered (the high pass filtering at 2 Hz was used to eliminate the big fluctuations related to voluntary pronation-supination movements during the buzz wire task (goal-directed kinetic tremor) (Budini et al. 2014b). Tremor was analysed in both time and frequency domains. For the time domain we considered the standard deviation of the filtered signal calculated for each of the three axes and tremor was quantified by computing the average of standard deviations of the three axes. For the frequency domain, tremor was quantified by the maximal value of the dominant peak within the physiological tremor frequency band (6–13 Hz) in the power spectra (2048-point, hamming window fast Fourier transform) of the accelerometer signals.

Muscular activation during the hand postural task was quantified in terms of root mean square (RMS) of the EMG signal for the entire 300 s contraction. Additionally, to check for variations in muscle activity during the 300 s contraction time that might have suggested onset of muscle fatigue, EMG RMS was also calculated for the first 30 s (from 0 to 30 s) and compared to the EMG RMS of the last 30 s (from 270 to 300 s).

Hand dexterity was assessed as number of touches and the contact time between the wand and the buzz wire circuit (Fig. 2) (Budini et al. 2017). Additionally, the number of pronation-supination movements (number of loops in the buzz wire circuit) accomplished during the 300 s were counted to verify whether the volunteers changed their speed when executing the kinetic assessment task between control and intervention conditions. Finger dexterity was assessed as the time to complete a Purdue Pegboard Assembly test.

### Statistical analysis

Data distribution was checked by Shapiro–Wilk test. In case data was normally distributed, a two-tailed paired *t* test was used to test between the conditions (intervention-n-back counting task vs control—no cognitive task), otherwise a Wilcoxon signed ranks test was used. The variables tested by adopting this statistical approach were: number of touches, contact time and number of loops completed during the 300 s the buzz wire test; total time to complete the Purdue Pegboard Assembly test (on a subsample of 13 participants); standard deviation (SD) of the acceleration (time domain) and position and amplitude of the dominant peak (after frequency domain analysis) of the acceleration during the hand postural and goal-directed kinetic (buzz wire) tasks; and the EMG RMS during the total 300 s hand postural task.

Differences between EMG RMS in the first and last 30 s for the two conditions (intervention vs control) during the hand postural task were analysed with an ANOVA for repeated measures (data was normally distributed) with two factors and two levels: intervention (control/effort) and time (first/last 30 s).

In the subgroup of six participant on which we conducted measurements at baseline, intervention, and immediately after intervention, data was normally distributed and there was homogeneity of variance and covariance between samples. Consequently, we used a repeated measures ANOVA test with a single factor and three levels (pre/intervention/post) followed by LSD post hoc tests for pairwise comparisons.

## Results

### Goal-directed kinetic tremor and hand dexterity

During the 300 s goal-directed kinetic tremor task (buzz wire), the volunteers completed 142.3 ± 38.3 loops in the control condition and 139.3 ± 41.0 during the cognitive effort (*t* = 0.447, p = 0.66). Despite this comparable task execution speed, the total number of touches and the contact time increased significantly from 8 ± 9 touches and 269 ± 290 ms contact time during control to 13 ± 13 touches and 603 ± 861 ms contact time during the cognitive task (*Z* = − 2.94, *p* = 0.003 and *Z* = − 2.25, *p* = 0.024, respectively).

**Fig. 3:**
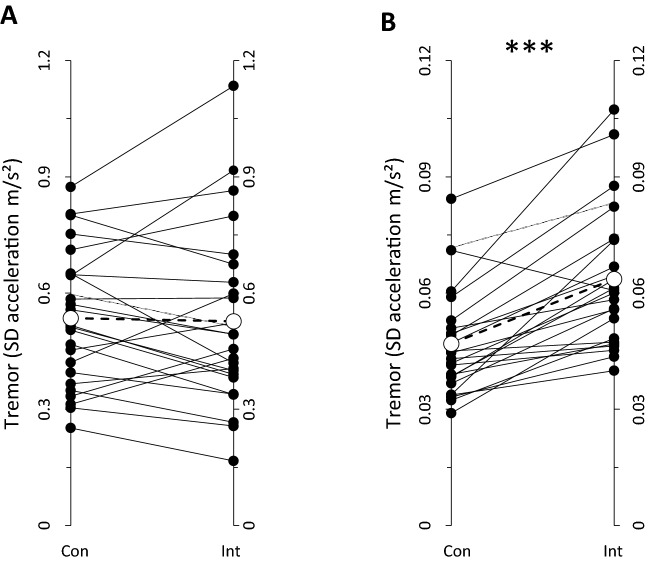
Tremor (as assessed by average standard deviation from acceleration axes) during 300 s continue goal-directed kinetic task (**A**) and hand postural task (**B**). Filled circles with solid lines represent individual subjects. Open circles with dashed line represent mean values. ***p < .0001

**Fig. 4:**
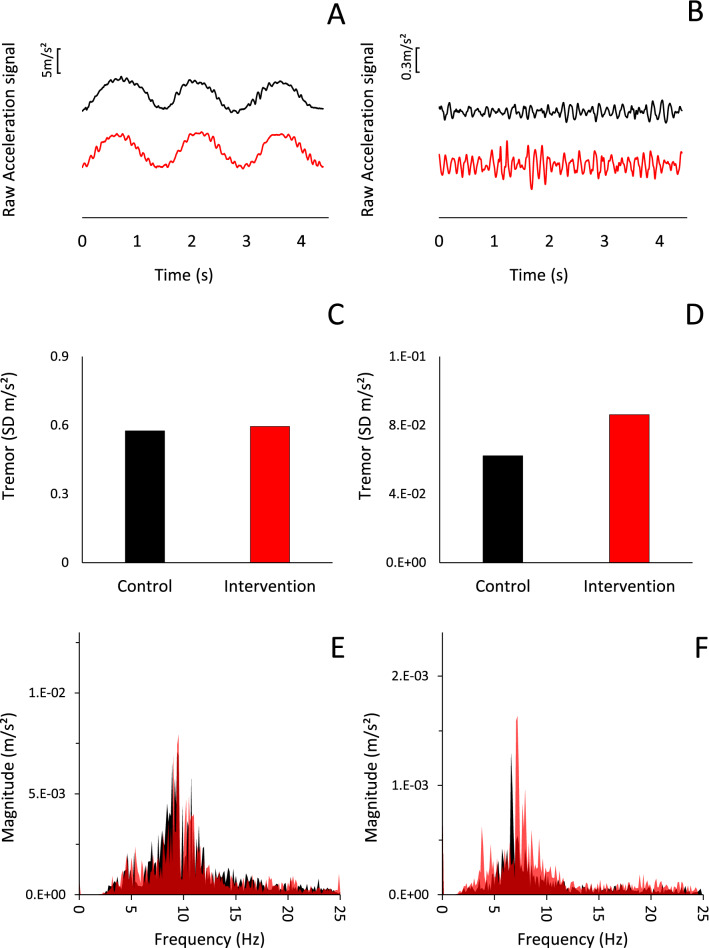
Raw and processed acceleration data from one representative subject for the Y axis. Left column: buzz wire task (goal-directed kinetic tremor); right column: hand postural task. Black: during control condition; red: during cognitive effort. **A**, **B**: 4.5 s raw acceleration data on the Y axis. **C**, **D**: Standard deviation of the acceleration on Y axis calculated on the entire 300 s duration of the task and **E**,**F**: the related analysis of the frequency domain. Black corresponds to control; red corresponds to intervention, and the darker red areas in **E**, **F** correspond to the areas where the fast Fourier transform plots of the control and intervention overlap. It can be noticed that in **E** the two areas overlap almost perfectly, whilst in **F** the size of the area corresponding to the intervention is greater than the one representing the control throughout the frequency spectrum

All the values related to the analysis of the acceleration data in the time and frequency domain are reported in Table 1. Tremor was not affected by the cognitive effort: the SD of the acceleration signal (3 axes averaged) (Fig. 3A), and the size and the position of the dominant peak for each axis within the tremor frequency band (Table 1) did not change (as shown for a representative subject in Fig. 4E) (*p* > 0.05 for all comparisons).

**Table 1:** Goal-directed kinetic task (buzz wire)

Kinetic task	SD acceleration (m/s^2^)								Main frequency amplitude (m/s^2^)								Main frequency position (Hz)							
	X		Y		Z		AV		X		Y		Z		AV		X		Y		Z		AV	
	Con	Int	Con	Int	Con	Int	Con	Int	Con	Int	Con	Int	Con	Int	Con	Int	Con	Int	Con	Int	Con	Int	Con	Int
	.89 ± .27	0.89 ± .36	.36 ± .14	.35 ± .18	.35 ± .15	.34 ± .17	.54 ± .17	.53 ± .23	.017 ± .013	.019 ± .021	.002 ± .002	.002 ± .002	.003 ± .003	.002 ± .003	.007 ± .006	.008 ± .009	10.0 ± .8	10.1 ± .8	9.8 ± .8	9.7 ± 1.0	9.9 ± 1.0	9.9 ± 1.1	9.9 ± .8	9.9 ± .9
	Percentage of average change						− 1.5%								+ 5.3%								− 0.5%

**Table 2:** Hand postural task

Postural task	SD acceleration (m/s^2^)								Main frequency amplitude (m/s^2^)								Main frequency position (Hz)							
	X		Y		Z		AV		X		Y		Z		AV		X		Y		Z		AV	
	Con	Int	Con	Int	Con	Int	Con	Int	Con	Int	Con	Int	Con	Int	Con	Int	Con	Int	Con	Int	Con	Int	Con	Int
	.06 ± .03	.07 ± .02	.06 ± .02	.08 ± .03	.03 ± .01	.04 ± .01	.05 ± .02	.06 ± .02	4E-5 ± 2E-5	7E-5 ± 8E-5	14E-5 ± 11E-5	24E-5 ± 32E-5	1E-5 ± 1E-5	2E-5 ± 3E-5	6E-5 ± 4E-5	11E-5 ± 13E-5	7.3 ± 1.5	7.0 ± 1.0	7.3 ± .7	7.4 ± .8	7.2 ± 1.2	7.3 ± .9	7.3 ± .7	7.3 ± .6
	Percentage of average change						+ 29.2%^a^								+ 79.0%^b^								− 0.4%	

In both tables the results for analyses of the time domain (standard deviation) and frequency domain (amplitude and position of the main peak) are reported. Values are detailed for each axis of the accelerometer. Average of the three axes and average percentage variation is also presented

*SD* Standard deviation, *X* *Y* *Z* Respective accelerometer axis, *AV* Average, *Con* control, *Int* Intervention

^a^*p* < .00001; ^b^*p* < .05

### Hand postural tremor

As depicted in Figs. 4B, 4D, and 4F for a representative participant, the size of the oscillations during the hand postural task increased while performing mental calculation. We observed this same result in 24 out of 25 participants (Fig. 3B). The group average SD of the acceleration signal (average of the 3 axes), is in line with the data of the representative subject, resulting in a significant increase during the cognitive effort (*Z* = − 4.086, *p* = 0.00004) (Fig. 3B, Table 2). This effect was mostly confined within the tremor frequency band since the increased SD mirrored an increase in the main peak amplitude (*Z* = − 2.200, *p* = 0.028), whilst the position of the main peak in the power spectrum analysis of the oscillation was not affected (*t* = 0.375, *p* = 0.71) (Fig. 4F for representative subject, Table 2 for group average).

This increased instability was not accompanied by variation in the amplitude of the 300 s average EMG RMS signal that did not show significant differences between control (0.059 ± 0.034 mV) and intervention (0.062 ± 0.033 mV) (*Z* = 0.486, *p* = 0.63). Likewise, no differences were observed in the first and last 30 s analysis for which no effect for time (first vs last, *F* = 0.010, *p* = 0.921), intervention (control vs effort *F* = 0.488, *p* = 0.493) or interaction (time*control *F* = 0.967, *p* = 0.337) were observed.

### Purdue pegboard test

A Purdue test was performed on a subgroup of 13 participants and analysed with a paired *t* test. The group average time to complete the test increased from 225 ± 18 s during the control condition to 253 ± 26 s during mental calculation (*t* = − 6.145, *p* = 0.00005).

### Lasting effect

**Fig. 5:**
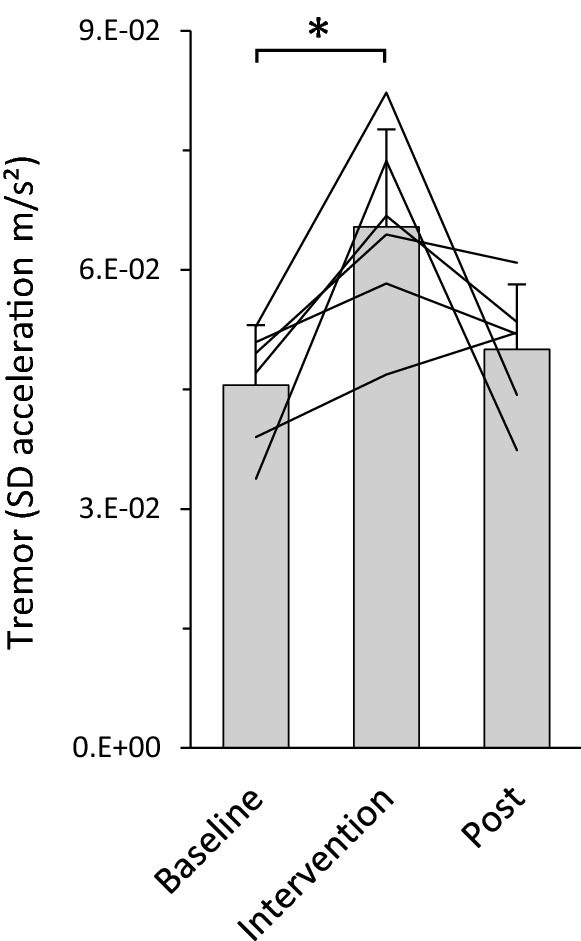
Tremor (assessed as standard deviation of mean accelerations) during the hand postural task calculated at baseline, during the intervention, and immediately after it. Bars represent the group average + standard deviation; individual subjects are reported with the lines. *p < .05

Lasting effect of the procedure was tested on a subsample of six participants and analysed with repeated measures ANOVA. Figure 5 shows the group average and individual subjects’ values for the SD of the acceleration signal during the hand postural task at baseline, during mental calculation, and immediately after it. In all subject there was an increase in the amplitude of the oscillation during the intervention that decreased again, in five out of six participants, after the termination of the cognitive task. Repeated measures ANOVA was significant (*F* = 7.152, *p* = 0.012) with pairwise comparisons highlighting differences between baseline and intervention (*p* = 0.013) but not between baseline and post (*p* = 0.222), or intervention and post (*p* = 0.089).

## Discussions

This study was conducted to investigate the effects of a cognitive effort on hand postural and goal-directed kinetic tremor. The results demonstrate an increase in tremor during the hand postural, but not during the kinetic task.

The simultaneous execution of two different tasks (cognitive and physical) leads to a well-known phenomenon reported as cognitive–motor interference with consequent reduction in performance in one or both tasks. It is therefore not surprising, and in agreement with previous works (Duncan et al. 2015; Rozand et al. 2015), that in our study both finger and hand dexterity resulted in lower scores when mental calculation was added compared to the control condition. Physiological muscle tremor, however, is not something that can be volitionally controlled (Marshall and Walsh 1956); consequently, it should not be susceptible of dual-task interference, at least not in relation to the psychological refractory period effect.

Because the mental effort induced a different response on postural and kinetic tremor, any mechanism that could have caused a general common response for both tasks, as for example a change in the hormonal status (Frankenhaeuser and Johansson 1976; Miki et al. 1998) should be ruled out, whilst an attempt should be done in linking the results to the different origins and characteristics of postural and kinetic physiological tremor.

The origin of physiological tremor has been investigated for over a century (Horsley and Schäfer 1886; Schäfer 1886); however, debate still exists about the contributions of central (Vallbo and Wessberg 1993; Bye and Neilson 2010) and peripheral (Joyce and Rack 1974; Vernooij et al. 2013) components, likely being both involved (Marsden 1984; Elble 1996). Our results on hand postural tremor indirectly support the position of the existence of a central contribution to physiological tremor. Indeed, if tremor was exclusively induced by peripheral mechanisms, then it should have not been affected by mental calculation. However, since the cognitive task we adopted required the verbal communication of the calculated result, it cannot be excluded that voice-induced vibrations transmitted to the limb have entrained with the ongoing physiological tremor, as suggested for ballisto-cardiographic effects (Awazu 1965; Tomonaga 1965), resulting in an increase in the amplitude of the oscillations. If this was the case, the dissimilar result between hand postural and goal-directed kinetic tremor could be attributed to a variation of the hand resonant component induced by a thixotropic effect during the kinetic movement and not during the hand postural task (Lakie et al. 2012; Vernooij et al. 2013). The possibility of an entrainment between physiological tremor and voice-induced vibration is, however, unlikely because, first, specific mechanical oscillation frequencies are required for that to happen (Halliday and Redfearn 1956; Lippold 1970; Joyce and Rack 1974), and second, while tremor frequency is very similar between different adult individuals (Marshall and Walsh 1956; Lakie 1995), a great variability exists in fundamental voice frequency between subjects (Atkinson 1976). Consequently, an entrainment (if any) could have occurred only in a percentage of our volunteers, we observed instead an increase in hand postural tremor in all our participants.

Alternative explanations could be searched for among the central mechanisms contributing to muscle tremor, since also these can differ between postural and kinetic tasks. Indeed it has long been hypothesised that postural and kinetic tremor generate from different central command patterns (Marsden 1984; Vallbo and Wessberg 1993). Accordingly, tremor accelerometery was shown to be coherent with the cortical EEG recorded during postural, but not kinetic tremor (Mehta et al. 2014). Such corticomuscular coherence was observed more often during weak isometric contractions than during phasic movements (Marsden et al. 2001), whilst correlations between motor units and tremor were reported during slow dynamic wrist movements, but not during hand postural contractions (Kakuda et al. 1999). These differences in corticomuscular and corticotremor coherence between postural and kinetic tasks provide an interpretation of the potential mechanism underpinning the observed results. Indeed, cognitive efforts alter the activity in the motor cortex leading to an increase in electroencephalographical activity in both theta (4–8 Hz) and alpha (8–13 Hz) frequency bands (Lal and Craig 2002; Boksem et al. 2005; Tartaglia et al. 2008; Zhao et al. 2012). An increased cortical activity in the tremor frequency band can be the cause of an increase in tremor if the peripheral oscillations are coherent with the central oscillations, so we can hypothesise that this was the case during the hand postural and not during the goal-directed kinetic task.

An alternative explanation could be presented in relation to the onset of muscle fatigue that is well known to increase muscle tremor (Bousfield 1932). It could be hypothesised that fatigue was predominantly induced during the hand postural task, since this involved 5 min continuous contraction (although minimal) of the same muscles, whilst the goal-directed kinetic task, consisting in prono-supination movements, allowed alternating period of rest of the involved musculature. Since an onset in fatigue is related to an increase in EMG activity in the active muscle (Viitasalo and Komi 1977), to test whether fatigue occurred, we compared the amplitude of the extensor digitorum communis EMG between the control and the intervention condition as well as the EMG activity during the first 30 s (0–30 s) with the EMG activity during the last 30 s (270–300 s). As reported in the results, no differences were observed, so the hypothesis that the extensor digitorum communis fatigued during the hand postural task, can be ruled out.

Finally, when in the subsample of six participants, the measurements were repeated immediately after the termination of the mental task, the amplitude of the oscillations has already returned to baseline level (Fig. 5). This result supports our previous suggestion (Budini et al. 2022) that the neurophysiological alterations responsible for the increase in tremor, only persist for the duration of the cognitive effort.

## Conclusions

Our study showed that mental calculation increases hand postural tremor, but does not have an effect on goal-directed kinetic tremor. The electrical activity of the main agonist muscle did not change during the task suggesting that it did not get fatigued. We hypothesise that the increase in hand postural tremor can be attributed to central rather than peripheral mechanisms.

## Abbreviations

EMG: Electromyography
A/D converted: Analogue to digital converter
RMS: Root mean square
SD: Standard deviation
ANOVA: Analysis of variance

## References

[CR1] Atkinson JE (1976). Inter-and intraspeaker variability in fundamental voice frequency. J Acoust Soc Am.

[CR2] Awazu T (1965). Studies on human minor tremors. Jpn J Physiol.

[CR3] Boksem MA, Meijman TF, Lorist MM (2005). Effects of mental fatigue on attention: an ERP study. Cogn Brain Res.

[CR4] Bousfield W (1932). The influence of fatigue on tremor. J Exp Psychol.

[CR5] Brown DM, Graham JD, Innes KI (2020). Effects of prior cognitive exertion on physical performance: a systematic review and meta-analysis. Sports Med.

[CR6] Budini F, Lowery M, Durbaba R, De Vito G (2014). Effect of mental fatigue on induced tremor in human knee extensors. J Electromyogr Kinesiol.

[CR7] Budini F, Lowery MM, Hutchinson M (2014). Dexterity training improves manual precision in patients affected by essential tremor. Arch Phys Med Rehabil.

[CR8] Budini F, Laudani L, Bernardini S, Macaluso A (2017). Local vibration inhibits H-reflex but does not compromise manual dexterity and does not increase tremor. Hum Mov Sci.

[CR9] Budini F (2022). Tremor, finger and hand dexterity and force steadiness, do not change after mental fatigue in healthy humans. PLoS ONE.

[CR10] Bye RT, Neilson PD (2010). The BUMP model of response planning: intermittent predictive control accounts for 10 Hz physiological tremor. Hum Mov Sci.

[CR11] Chaudhuri A, Behan PO (2004). Fatigue in neurological disorders. Lancet.

[CR12] Deuschl G, Bain P, Brin M, Ad Hoc Scientific Committee (1998). Consensus statement of the movement disorder society on tremor. Mov Disord.

[CR13] Duncan MJ, Fowler N, George O (2015). Mental fatigue negatively influences manual dexterity and anticipation timing but not repeated high-intensity exercise performance in trained adults. Res Sports Med.

[CR14] Elble RJ (1996). Central mechanisms of tremor. J Clin Neurophysiol.

[CR15] Elble RJ (2003). Characteristics of physiologic tremor in young and elderly adults. Clin Neurophysiol.

[CR16] Fitts RH (1994). Cellular mechanisms of muscle fatigue. Physiol Rev.

[CR17] Fleishman EA, Ellison GD (1962). A factor analysis of fine manipulative tests. J Appl Psychol.

[CR18] Frankenhaeuser M, Johansson G (1976). Task demand as reflected in catecholamine excretion and heart rate. J Human Stress.

[CR19] Franz SI (1897). Six reviews of articles on mental fatigue and performance. Psychol Rev.

[CR20] Gandevia SC (2001). Spinal and supraspinal factors in human muscle fatigue. Physiol Rev.

[CR21] Halliday AM, Redfearn J (1956). An analysis of the frequencies of finger tremor in healthy subjects. J Physiol.

[CR22] Hopstaken JF, van der Linden D, Bakker AB (2016). Shifts in attention during mental fatigue: evidence from subjective, behavioral, physiological, and eye-tracking data. J Exp Psychol Hum Percept Perform.

[CR23] Horsley V, Schäfer E (1886). Experiments on the character of the muscular contractions which are evoked by excitation of the various parts of the motor tract. J Physiol.

[CR24] Ivanov BD, Kaprelyan AG, Bochev PH (2015). (18F)-FDG PET/CT in essential tremor: preliminary results. J IMAB Annu Proc Sci Pap.

[CR25] Joyce G, Rack PM (1974). The effects of load and force on tremor at the normal human elbow joint. J Physiol.

[CR26] Kakuda N, Nagaoka M, Wessberg J (1999). Common modulation of motor unit pairs during slow wrist movement in man. J Physiol.

[CR27] Lakie M, Vernooij CA, Osborne TM, Reynolds RF (2012). The resonant component of human physiological hand tremor is altered by slow voluntary movements. J Physiol.

[CR28] Lakie M (1995) Is essential tremor physiological? In: Findley LJ & Koller WC (eds) Handbook of Tremor Disorders, Marcel Dekker, New York, pp 165–183

[CR29] Lal SK, Craig A (2002). Driver fatigue: electroencephalography and psychological assessment. Psychophysiology.

[CR30] Langner R, Steinborn MB, Chatterjee A (2010). Mental fatigue and temporal preparation in simple reaction-time performance. Acta Psychol (amst).

[CR31] Lim J, Dinges D (2008). Sleep deprivation and vigilant attention. Ann N Y Acad Sci.

[CR32] Lim J, Wu W, Wang J (2010). Imaging brain fatigue from sustained mental workload: an ASL perfusion study of the time-on-task effect. Neuroimage.

[CR33] Lippold OC (1970). Oscillation in the stretch reflex arc and the origin of the rhythmical, 8–12 c/s component of physiological tremor. J Physiol.

[CR34] Lorist MM, Klein M, Nieuwenhuis S (2000). Mental fatigue and task control: planning and preparation. Psychophysiology.

[CR35] Lorist MM, Kernell D, Meijman TF, Zijdewind I (2002). Motor fatigue and cognitive task performance in humans. J Physiol.

[CR36] Lorist MM, Boksem MA, Ridderinkhof KR (2005). Impaired cognitive control and reduced cingulate activity during mental fatigue. Cogn Brain Res.

[CR37] Marsden C (1984). Origins of normal and pathological tremor. Movement disorders: tremor.

[CR38] Marsden JF, Brown P, Salenius S (2001). Involvement of the sensorimotor cortex in physiological force and action tremor. Neuro Report.

[CR39] Marshall J, Walsh EG (1956). Physiological tremor. J Neurol Neurosurg Psychiatry.

[CR40] Mehta AR, Brittain J-S, Brown P (2014). The selective influence of rhythmic cortical versus cerebellar transcranial stimulation on human physiological tremor. J Neurosci.

[CR41] Miki K, Kawamorita K, Araga Y (1998). Urinary and salivary stress hormone levels while performing arithmetic calculation in a noisy environment. Ind Health.

[CR42] Pageaux B, Lepers R (2018). The effects of mental fatigue on sport-related performance. Progress in brain research.

[CR43] Pereira HM, Schlinder-DeLap B, Keenan KG (2019). Oscillations in neural drive and age-related reductions in force steadiness with a cognitive challenge. J Appl Physiol.

[CR44] Rozand V, Lebon F, Papaxanthis C, Lepers R (2015). Effect of mental fatigue on speed–accuracy trade-off. Neuroscience.

[CR45] Sasahara I, Fujimura N, Nozawa Y (2015). The effect of histidine on mental fatigue and cognitive performance in subjects with high fatigue and sleep disruption scores. Physiol Behav.

[CR46] Schäfer E (1886). On the rhythm of muscular response to volitional impulses in man. J Physiol.

[CR47] Schöberl F, Feil K, Xiong G (2017). Pathological ponto-cerebello-thalamo-cortical activations in primary orthostatic tremor during lying and stance. Brain.

[CR48] Tartaglia M, Narayanan S, Arnold D (2008). Mental fatigue alters the pattern and increases the volume of cerebral activation required for a motor task in multiple sclerosis patients with fatigue. Eur J Neurol.

[CR49] Taylor JL, Gandevia SC (2008). A comparison of central aspects of fatigue in submaximal and maximal voluntary contractions. J Appl Physiol.

[CR50] Tomonaga K (1965). On the effect of heart beat on minor tremor. Jpn J Physiol.

[CR51] Valenza A, Charlier H, Bianco A, Filingeri D (2020). Independent and interactive effects of thermal stress and mental fatigue on manual dexterity. Am J Physiol-Regul Integr Comp Physiol.

[CR52] Vallbo A, Wessberg J (1993). Organization of motor output in slow finger movements in man. J Physiol.

[CR53] Van Cutsem J, Marcora S, De Pauw K (2017). The effects of mental fatigue on physical performance: a systematic review. Sports Med.

[CR54] Vanden Noven ML, Pereira HM, Yoon T (2014). Motor variability during sustained contractions increases with cognitive demand in older adults. Front Aging Neurosci.

[CR55] Vernooij CA, Reynolds RF, Lakie M (2013). A dominant role for mechanical resonance in physiological finger tremor revealed by selective minimization of voluntary drive and movement. J Neurophysiol.

[CR56] Viitasalo JH, Komi PV (1977). Signal characteristics of EMG during fatigue. Eur J Appl Physiol.

[CR57] Zhao C, Zhao M, Liu J, Zheng C (2012). Electroencephalogram and electrocardiograph assessment of mental fatigue in a driving simulator. Accid Anal Prev.

[CR58] Zijdewind I, van Duinen H, Zielman R, Lorist MM (2006). Interaction between force production and cognitive performance in humans. Clin Neurophysiol.

